# Comparison of pneumonitis risk between immunotherapy alone and in combination with chemotherapy: an observational, retrospective pharmacovigilance study

**DOI:** 10.3389/fphar.2023.1142016

**Published:** 2023-04-13

**Authors:** Huixia Li, Yifan Zheng, Peihang Xu, Zimu Li, Yukun Kuang, Xiaoqing Feng, Junhao He, Jia Li, Xiao Chen, Lihong Bai, Ke-Jing Tang

**Affiliations:** ^1^ Department of Pulmonary and Critical Care Medicine, The First Affiliated Hospital, Sun Yat-sen University, Guangzhou, China; ^2^ Department of Clinical Pharmacy Translational Science, College of Pharmacy, University of Michigan, Ann Arbor, MI, United States; ^3^ State Key Laboratory of Respiratory Disease, National Clinical Research Center for Respiratory Disease, Guangzhou Institute of Respiratory Health, The First Affiliated Hospital, Guangzhou Medical University, Guangzhou, China; ^4^ Department of Pulmonary and Critical Care Medicine, The Seventh Affiliated Hospital, Sun Yat-sen University, Shenzhen, China; ^5^ School of Pharmaceutical Sciences, Sun Yat-sen University, Guangzhou, China; ^6^ Department of Pharmacy, The First Affiliated Hospital, Sun Yat-sen University, Guangzhou, China

**Keywords:** immune-related adverse events (IRAE), chemotherapy, checkpoint inhibitor pneumonitis, immune checkpoint inhibitor (ICI), immunotherapy

## Abstract

**Importance:** Checkpoint inhibitor pneumonitis (CIP) is a rare but serious adverse event that may impact treatment decisions. However, there is limited information comparing CIP risks between immune checkpoint inhibitor (ICI) monotherapy and combination with chemotherapy due to a lack of direct cross-comparison in clinical trials.

**Objective:** To determine whether ICI combination with chemotherapy is superior to ICI in other drug regimens (including monotherapy) in terms of CIP risk.

**Study Design and Methods:** This observational, cross-sectional and worldwide pharmacovigilance cohort study included patients who developed CIP from the World Health Organization database (WHO) VigiBase and the US Food and Drug Administration Adverse Event Reporting System (FAERS) database. Individual case safety reports (ICSR) were extracted from 2015 to 2020 in FAERS and from 1967 to 2020 in VigiBase. Timing and reporting odds ratio (ROR) of CIP in different treatment strategies were used to detect time-to-onset and the risk of pneumonitis after different immunotherapy regimens.

**Results:** A total of 93,623 and 114,704 ICI-associated ICSRs were included in this study from VigiBase and FAERS databases respectively. 3450 (3.69%) and 3278 (2.86%) CIPs occurred after therapy initiation with a median of 62 days (VigiBase) and 40 days (FAERS). Among all the CIPs, 274 (7.9%) and 537 (16.4%) CIPs were associated with combination therapies. ICIs plus chemotherapy combination was associated with pneumonitis in both VigiBase [ROR 1.35, 95% CI 1.18-1.52] and FAERS [ROR 1.39, 95% CI 1.27–1.53]. The combination of anti-PD-1 antibodies and anti-CTLA-4 antibodies with chemotherapy demonstrated an association with pneumonitis in both VigiBase [PD-1+chemotherapy: 1.76, 95% CI 1.52-2.05; CTLA-4+chemotherapy: 2.36, 95% CI 1.67-3.35] and FAERS [PD-1+chemotherapy: 1.70, 95% CI 1.52-1.91; CTLA-4+chemotherapy: 1.70, 95% CI 1.31-2.20]. Anti-PD-L1 antibodies plus chemotherapy combinations did not show the association.

**Conclusion:** Compared to ICI in other drug regimens (including monotherapy), the combination of ICI plus chemotherapy is significantly associated with higher pneumonitis toxicity. Anti-PD-1/CTLA4 medications in combination with chemotherapy should be obviated in patients with potential risk factors for CIP.

Trial Registration: clinicaltrials.gov, ChiCTR2200059067

## 1 Introduction

Identifying and targeting the Achilles’ heel of various cancer has been an eternal task for oncologists. Chemotherapy, discovered empirically in the 1950-70s as the first therapeutic arsenal, was once a star in cancer treatment. By targeting and inhibiting highly proliferating cells, it pioneered survival prolongation for cancer patients as the first pharmacological answer. However, for most prevalent solid malignancies at metastatic stages, chemotherapy only offers modest overall survival benefits. Furthermore, the induced resistance and significant toxicity from chemotherapy devalue its maximum clinical benefit (Feliu et al., 2020; Ippolito et al., 2021). Immune checkpoint inhibitors (ICIs), either alone or combined with chemotherapy, have revolutionized the treatment landscape of multiple types of advanced cancer in recent years. By interacting with programmed cell death 1/ligand 1(PD-1/PD-L1) and cytotoxic T lymphocyte-associated antigen 4 (CTLA-4), ICIs prevent cancers from escaping from antitumor immune response, leading to significant improvement in clinical outcome (Dafni et al., 2019; Davern and Lysaght, 2020; Galluzzi et al., 2020).

However, immune-related adverse events (irAEs) that stem from the activation of autoreactive T cells attacking host tissues often necessitate changes in or discontinuation of the treatment regimen and pose a risk of morbidity. Among these irAEs, pneumonitis is the most common fatal pulmonary adverse event with a mortality rate of up to 20% (Wang et al., 2018; Suresh et al., 2019a). About 1%–4% of patients on ICI monotherapy and 4%–7% on combination developed pneumonitis (Delaunay et al., 2017; Cadranel et al., 2019). Several studies have described pneumonitis derived from ICIs in clinical trials (Chen et al., 2020). Nevertheless, these studies lack direct cross-comparison and most of them were unable to comprehensively characterize them due to the small incidence and sample size. Furthermore, data from clinical trials may not be representative of the real-world population and settings.

With solid evidence from clinical trials appearing, ICI plus chemotherapy has become one of the first-line treatment regimens for various tumors (Dolladille et al., 2020). The need to understand the pneumonitis risks of these combinations is critical as it is an important factor influencing treatment decision-making. However, to the best of our knowledge, few studies characterized pneumonitis from the combination of immune checkpoint inhibitors plus chemotherapy (chemotherapy use before or concomitant with ICIs) and controversy existed from various clinical trial meta-analyses (Xu et al., 2018; Chen et al., 2020). How the combination may impact pneumonitis toxicity in the real world remains a key unknown clinical topic (Sears et al., 2019). Herein, we used the World Health Organization (WHO) pharmacovigilance database (VigiBase) and the US Food and Drug Administration (US FDA) Adverse Event Reporting System (FAERS) database to extensively characterize and compare the pneumonitis risk from the ICI alone and the combinations of ICI plus chemotherapy.

## 2 Materials and methods

### 2.1 Study design and data sources

This observational, retrospective, pharmacovigilance study is a disproportionality analysis based on the individual case safety reports (ICSRs) extracted from both VigiBase and FAERS. VigiBase is one of the largest pharmacovigilance databases managed by the WHO Uppsala Monitoring Centre (Uppsala, Sweden). It includes more than 21 million ICSRs from more than 130 participating countries since 1968 (as of 2020/8/30). The WHO ICSRs were collected from various sources including healthcare professionals, patients, and pharmaceutical companies and most of them are post-marketing data. FAERS is also an important source of post-marketing safety surveillance for all approved drug and therapeutic biologic products worldwide, and it consists of more than 8 million subject reports from 2015 to 2020. Therefore, the size and worldwide coverage of these two databases make them particularly robust for the conduction of spontaneous reporting data analysis. The use of confidential, electronically processed patient data was approved by our Institutional Review Board by our institution’s ethics committee and this study was registered as a clinical trial at ChiCTR2200059067.

### 2.2 VigiBase and FAERS procedures

The pharmacovigilance study included all reports of pneumonitis irAEs and two preferred terms (PTs): “pneumonitis” and “immune-mediated pneumonitis” were selected based on expert consensuses according to the Medical Dictionary for Regulatory Activities (MedDRA). Eight ICI drugs were investigated and discussed in this study, including anti-PD-1 antibodies (nivolumab, pembrolizumab, and cemiplimab), anti-PD-L1 antibodies (atezolizumab, avelumab, and durvalumab), and anti-CTLA-4 antibodies (ipilimumab and tremelimumab). 76 cytotoxic chemotherapeutic drugs were selected as chemotherapy in this study based on a review from Bailly et al. (Supplementary Appendix S1).

In the VigiBase database, ICSRs include patient demographics information (sex and age), general administrative information (reporting country, reporting date, and notifier qualification), drug information (indication, regimen, route of administration), and reactions (time to onset, seriousness, outcome, MedDRA preferred term). However, not all ICSRs contain complete information. In the FAERS database, 5 datasets were drawn together, in which demographic information (DEMO), drugs with dates of start and end (DRUG and THER), irAEs and their outcomes (REAC and OUCT), and indications of use (INDI) are contained. The ICSRs assessed in the study were those notified as ICIs suspected/interacting and chemotherapeutic drugs suspected/interacting/concomitant events.

### 2.3 Statistical analysis

Adapting previous methods published in influential journals (Oshima et al., 2018; Salem et al., 2018; Contejean et al., 2021; Reese et al., 2021), we conducted a disproportionality analysis (known as case-non-case analysis) to assess whether suspected pneumonitis irAEs were differentially reported with a combination of ICI plus chemotherapy compared with ICI in other drug regimens, including monotherapy. We calculated the reporting odds ratio (ROR) and 95% CI in patients who received the ICI plus chemotherapy *versus* ICI in other drug regimens (including monotherapy). The ROR disproportionality analysis has been one of the frequentist measures of association, adapted from the contingency table of the drug combination and counts of adverse event reports. The ROR (95% CI) was calculated $\frac{ad}{bc}\left[e^{\pm 1.96\sqrt{\frac{1}{a}+\frac{1}{b}+\frac{1}{c}+\frac{1}{d}}}\right]$ , where *a* is the number of pneumonitis irAEs cases exposed to the ICI plus chemotherapy combination, *b* is the number of non-cases exposed to the ICI plus chemotherapy combination, *c* is the number of pneumonitis group cases to the comparator (other ICI-containing regimens) and *d* is the number of non-cases exposed to the comparator. When the lower bound of ROR 95% CI is greater than 1.00, it implies that a significant disproportionality signal was detected and can be interpreted as statistically more adverse events observed for the drug/drug combination than one would except by chance alone. All the analyses were performed on R studio (Version 4.0.2). Means, standard deviations (SD), and median were calculated for adverse reaction demographic description if necessary. The significance level was set at *p* < 0.05. Time to irAEs of the pneumonitis group was evaluated using the Kaplan-Meier method and compared using the log-rank test.

## 3 Results

### 3.1 Demographics description

In the VigiBase and FAERS databases, a total of 93,623 and 114,704 ICI-associated ICSRs were included in this study respectively. Among all the ICSRs, 3,450 and 3,278 pneumonitis irAEs group cases were captured respectively in VigiBase and FAERS. In both databases, we noticed consistent distributions in reporting regions, reporters, reporting year, sex, age at onset, and indication. Most cases were reported from the Americas and Eastern Mediterranean Europe between 2017-2020. Healthcare professionals are the main reporters. Most reported pneumonitis cases were in male patients. On average of the two databases, around 14% of pneumonitis were fatal. Approximately half of all patients who had pneumonitis were treated with ICIs for lung cancer. In the FAERS database, 16.4% of pneumonitis reports (n = 537) were associated with the combinations of ICI plus chemotherapy, while in the VigiBase, 7.9% (n = 274) of the reports were associated with the combination therapies. Table 1 lists the study population demographic description.

**TABLE 1:** Characteristics of patients with CIP from VigiBase and FAERS databases.

	WHO			FDA		
	ICI without chemotherapy	ICI with chemotherapy	Total	ICI without chemotherapy	ICI with chemotherapy	Total
**Case N (%)**	3176	274	3450	2741	537	3278
**Reporting region**						
African	9 (0.28%)	3 (1.09%)	12 (0.35%)	9 (0.33%)	3 (0.56%)	12 (0.37%)
Eastern Mediterranean European	1291 (40.65%)	78 (28.47%)	1369 (39.68%)	764 (27.87%)	156 (29.05%)	920 (28.07%)
Americas	1233 (38.82%)	163 (59.49%)	1396 (40.46%)	1450 (52.9%)	265 (49.35%)	1715 (52.32%)
South East Asian Western Pacific	643 (20.25%)	30 (10.95%)	673 (19.51%)	512 (18.68%)	110 (20.48%)	622 (18.92%)
Unknown	0	0	0	6 (0.22%)	3 (0.56%)	9 (0.27%)
Reporters*						
Healthcare professional	2807 (80.57%)	247 (79.42%)	3054 (80.47%)	2072 (75.59%)	430 (80.07%)	2502 (76.33%)
Non-healthcare professional	536 (15.38%)	58 (18.65%)	594 (15.65%)	619 (22.58%)	104 (19.37%)	723 (22.06%)
Unknown	141 (4.05%)	6 (1.93%)	147 (3.87%)	50 (1.82%)	3 (0.56%)	53 (1.62%)
**Reporting year**						
2020	364 (11.46%)	68 (24.82%)	432 (12.52%)	329 (12%)	110 (20.48%)	439 (13.39%)
2019	1075 (33.85%)	94 (34.31%)	1169 (33.88%)	770 (28.09%)	199 (37.06)	969 (29.56%)
2018	780 (24.56%)	72 (26.28%)	852 (24.70%)	756 (27.58%)	135 (25.14%)	891 (27.18%)
2017	552 (17.38%)	24 (8.67%)	576 (16.7%)	452 (16.49%)	56 (10.43%)	508 (15.5%)
2016	248 (7.81%)	11 (4.01)	259 (7.51%)	290 (10.58%)	25 (4.66%)	315 (9.61)
2015 and before	157 (4.84%)	5 (1.82%)	162 (4.70%)	144 (5.26%)	12 (2.23%)	156 (4.76%)
**Sex**						
Male	1926 (60.64%)	104 (37.96%)	2030 (58.84%)	1594 (58.15%)	291 (54.19%)	1885 (57.5%)
Female	923 (29.06%)	141 (51.46%)	1064 (30.84%)	815 (29.73%)	188 (35.01%)	1003 (30.6%)
Unknown	327 (10.30%)	29 (10.58%)	356 (10.32%)	332 (12.11%)	58 (10.8%)	390 (11.9%)
**Age at onset**						
<18 years	1 (0.03%)	0	1 (0.03%)	3 (0.11%)	1 (0.19%)	4 (0.12%)
18–44 years	111 (3.49%)	7 (2.55%)	118 (3.42%)	132 (4.82%)	12 (2.23%)	144 (4.39%)
45–64 years	774 (24.37%)	90 (32.85%)	864 (25.04%)	740 (27%)	200 (37.24%)	940 (28.68%)
65–74 years	755 (23.77%)	72 (26.28%)	827 (23.97%)	708 (25.83%)	175 (32.59%)	883 (26.94%)
>74 years	388 (12.22%)	34 (12.41%)	422 (12.23%)	347 (12.66%)	43 (8.01%)	390 (11.9%)
unknown	1147 (36.11%)	71 (25.91%)	1218 (35.30%)	811 (29.59%)	106 (19.74%)	917 (27.97%)
**Time to ADR onset (median IQR)**	61.75 (21–120.47)	74 (21–106.4)	62 (21–119.5)	41 (14–105)	37 (12–117)	40 (13–109)
**Outcome**						
Fatal	285 (8.95%)	37 (13.50%)	324 (9.37%)	493 (18.0%)	493 (18.0%)	614 (18.7%)
Non-fatal	1556 (48.85%)	141 (51.45%)	1691 (48.89%)	2175 (79.3%)	121 (22.5%)	2585 (78.9%)
Unknown	1344 (42.20%)	96 (35.0%)	1444 (41.75%)	73 (2.66%)	6 (1.1%)	79 (2.41%)
**Indication**						
Lung cancer	1713 (45.33%)	132 (39.88%)	1845 (44.89%)	1281 (46.0%)	278 (51.58%)	1559 (46.9%)
Melanoma	647 (17.12%)	13 (3.93%)	660 (16.06%)	512 (18.38%)	10 (1.86%)	522 (15.7%)
Gastrointestinal carcinoma	74 (1.96%)	21 (6.34%)	95 (2.31%)	102 (3.66%)	51 (9.46%)	153 (4.6%)
Hematologic cancer and lymphoma	76 (2.01%)	52 (15.71%)	128 (3.11%)	97 (3.48%)	67 (12.43%)	164 (4.93%)
Renal cell carcinoma	195 (5.16%)	0	195 (4.74%)	179 (6.43%)	1 (0.19%)	180 (5.42%)
Urothelia carcinoma	63 (1.67%)	10 (3.02%)	73 (1.78%)	50 (1.8%)	13 (2.41%)	63 (1.9%)
Breast cancer	23 (0.61%)	20 (6.04%)	43 (1.05%)	25 (0.9%)	33 (6.12%)	58 (1.74%)
Cancer of head and neck	58 (1.53%)	2 (0.60%)	60 (1.46%)	62 (2.23%)	5 (0.93%)	67 (2.02%)
Others/Unknow	930 (24.61%)	81 (24.47%)	1011 (24.60%)	477 (17.13%)	81 (15.03)	558 (16.79%)

**FIGURE 1:**
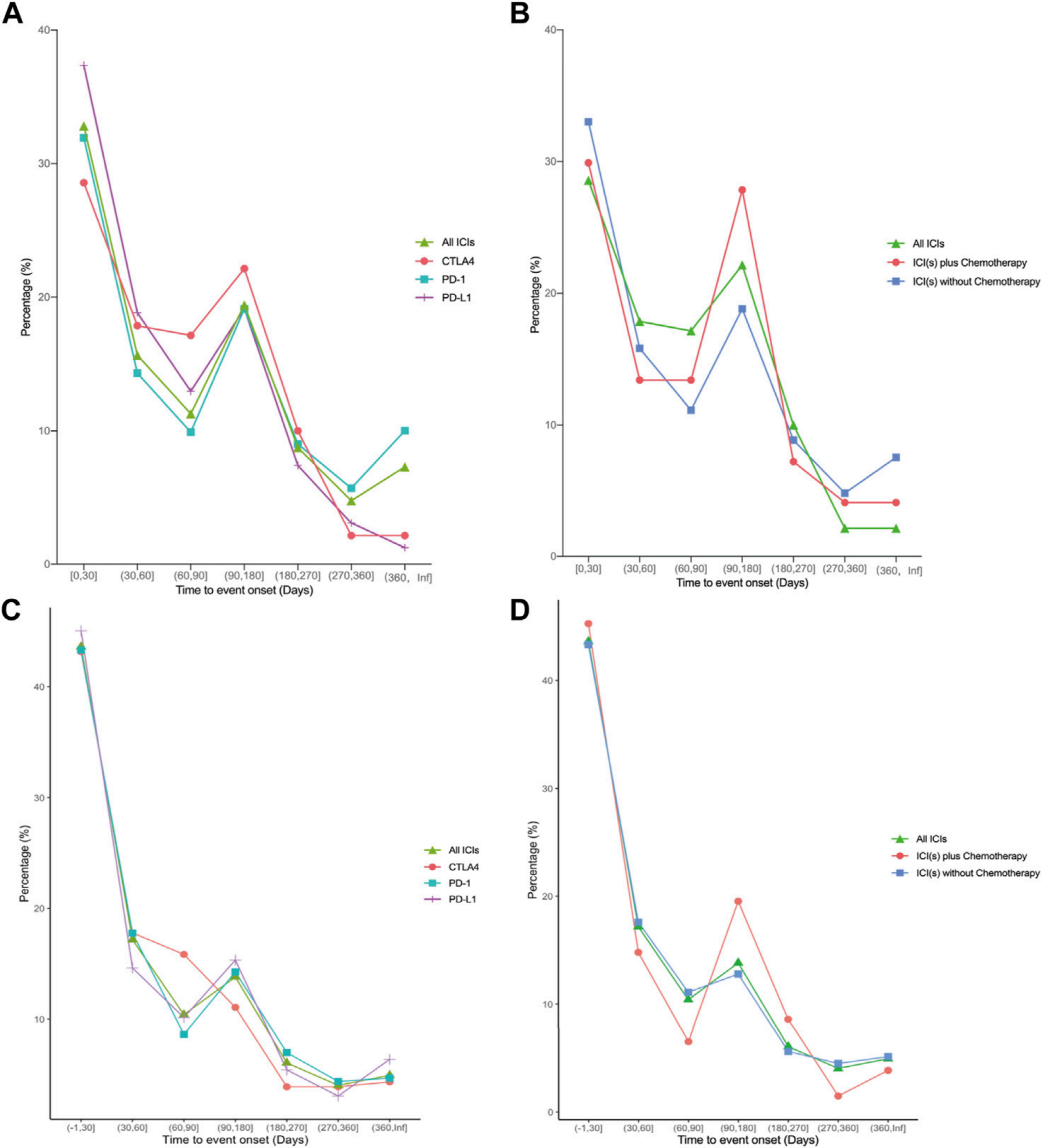
Line Plot depicting time-to-onsets of ICI-related pneumonitis after different treatment regimens of ICI(s). **(A–B)** data from the VigiBase database. **(C–D)** data from the FAERS database. ICI: immune checkpoint inhibitor; PD-1: programmed cell death receptor 1 inhibitors; PD-L1: programmed cell death ligand 1 inhibitors; CTLA4: cytotoxic T lymphocyte antigen 4 inhibitors.

The regimen-specific timing of these irAEs was compared between treatment types (Figure 1). The median time to adverse event onset occurred early after therapy onset, either immunotherapy alone or in combination with chemotherapy (41 days vs. 37 days in FAERS, *p* < 0.05; 61.5 days vs. 74 days in VigiBase, *p* > 0.05).

**FIGURE 2:**
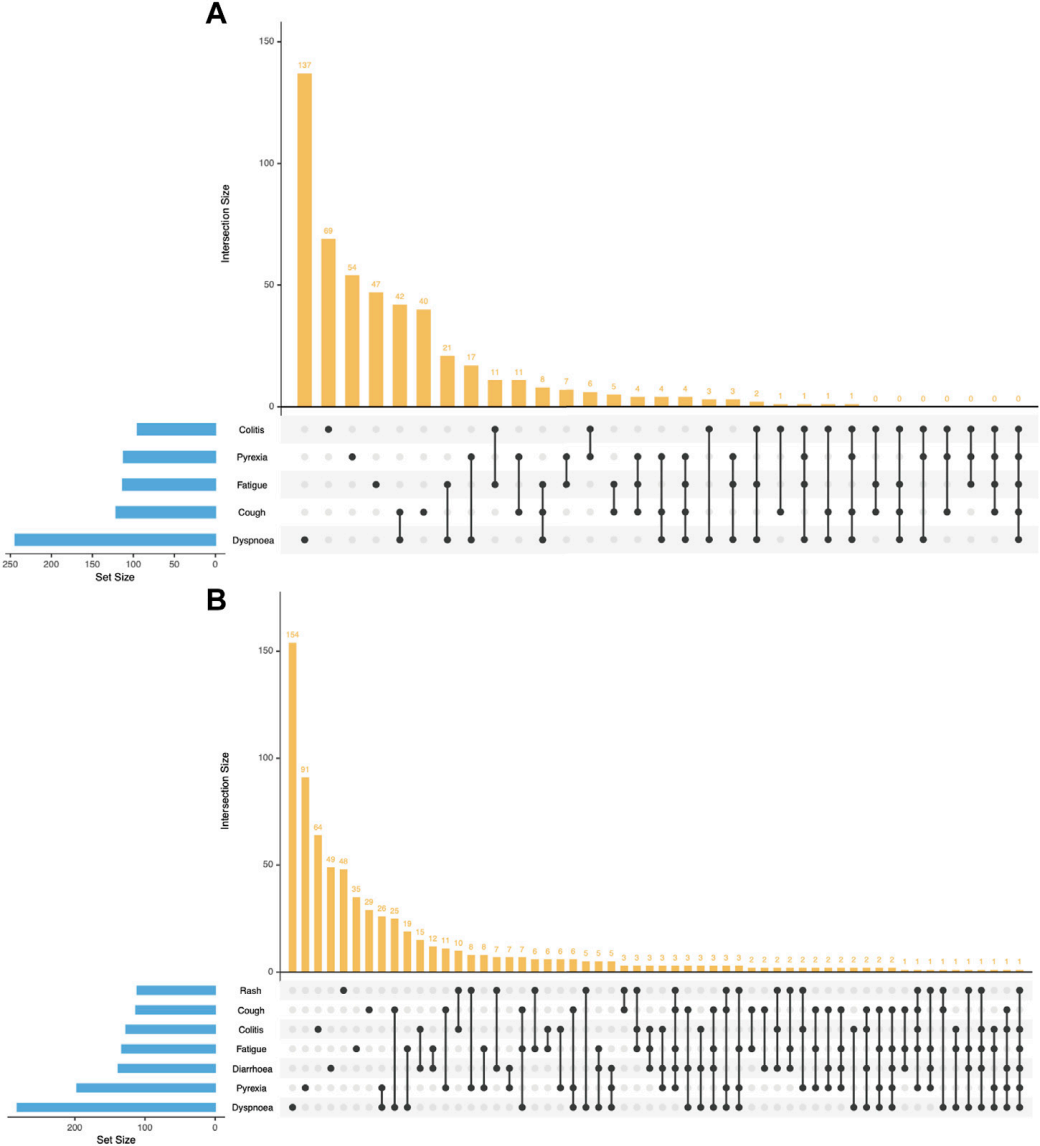
UpSet Plot depicting top 5 concomitant toxicities along with pneumonitis in VigiBase and top 7 concomitant toxicities with FAERS databases. **(A)** data from the VigiBase database. **(B)** data from the FAERS database.

Many other toxicities happened along with pneumonitis. Figure 2 showed the top 5 concomitant toxicities in VigiBase and the top 7 in FAERS. In VigiBase, the top 5 concomitant toxicities were similar, including dyspnea, cough, fatigue, pyrexia and colitis. The top 7 concomitant toxicities along with pneumonitis in FAERS were dyspnea, pyrexia, diarrhea, fatigue, colitis, cough and rash. In both databases, we noticed dyspnea as the most common concomitant toxicity.

### 3.2 Disproportionality analysis

**FIGURE 3:**
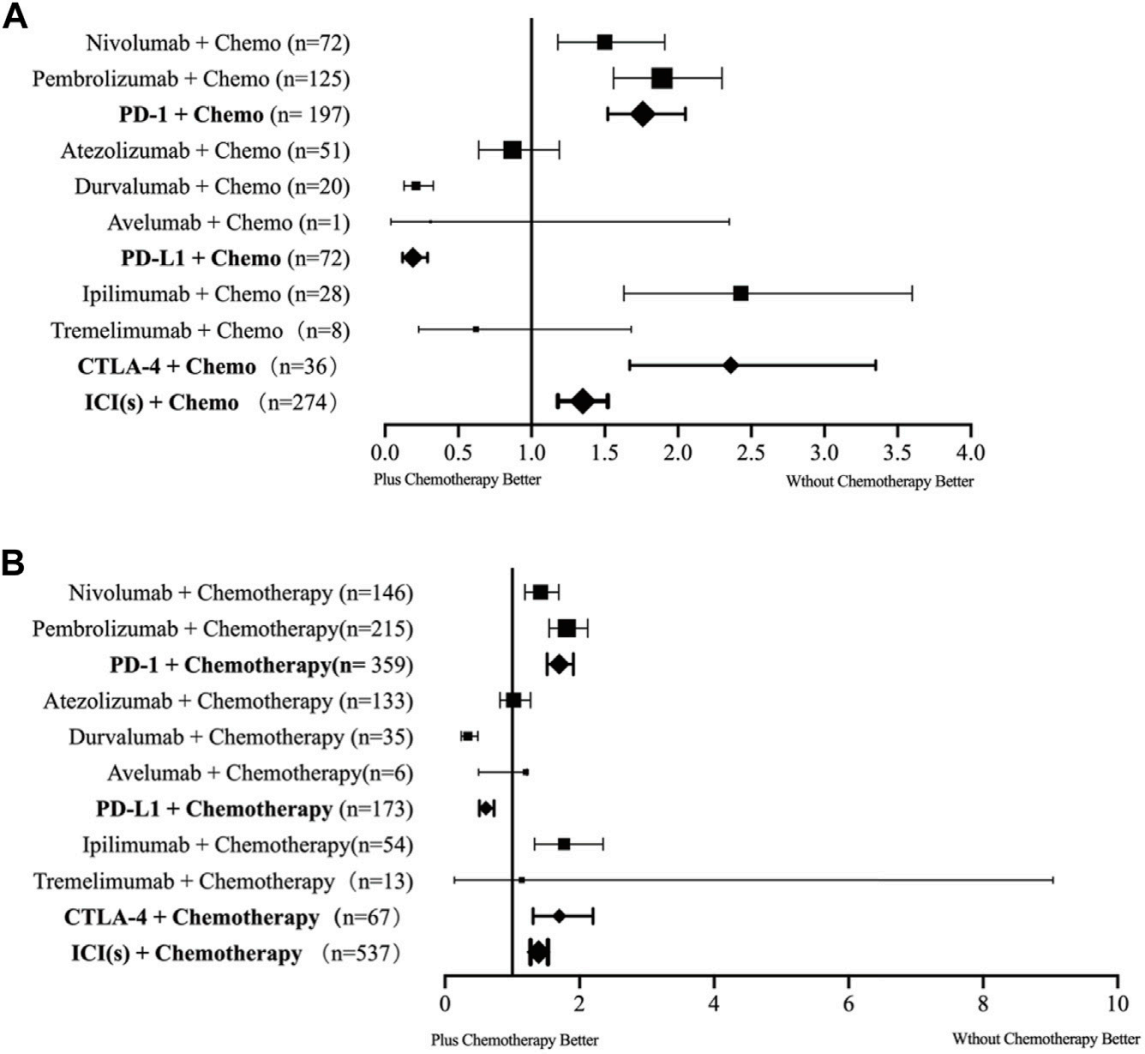
Forest plot of the incidence rates of checkpoint inhibitor pneumonitis for each type of combination therapy. **(A)** data from the VigiBase database. **(B)** data from the FAERS database. Chemo: chemotherapy; ICI: immune checkpoint inhibitor; PD-1: programmed cell death receptor 1 inhibitors; PD-L1: programmed cell death ligand 1 inhibitors; CTLA4: cytotoxic T lymphocyte antigen 4 inhibitors.

ICSRs were evaluated in the FAERS and VigiBase databases based on two MedDRA search terms as mentioned previously (“pneumonitis” and “immune-mediated pneumonitis”). Table 2 and Figure 3 showed the disproportionality analysis results from the two databases.

**TABLE 2:** The disproportionality analysis of patients with ICI plus chemotherapy and ICI in other drug regimens in VigiBase and FAERS databases.

	VigiBase			FAERS		
	Cases	Non-cases	ROR (95% CI)	Cases	Non-cases	ROR (95% CI)
Nivolumab + Chemotherapy	72	1408	1.50 (1.18–1.91)	146	3992	1.42 (1.19–1.69)
Pembrolizumab + Chemotherapy	125	1821	1.89 (1.56–2.30)	215	3916	1.81 (1.55–2.12)
**PD-1 + Chemotherapy**	**197**	**3220**	**1.76 (1.52–2.05)**	**359**	**7,902**	**1.70 (1.52–1.91)**
						
Atezolizumab + Chemotherapy	51	1275	0.87 (0.64–1.19)	133	4,119	1.02 (0.82–1.27)
Durvalumab + Chemotherapy	20	350	0.21 (0.13–0.33)	35	798	0.34 (0.24–0.49)
Avelumab + Chemotherapy	1	173	0.31 (0.04–2.35)	6	463	0.5 (1.2–1.22)
**PD-L1 + Chemotherapy**	**21**	**522**	**0.19 (0.12–0.29)**	**173**	**5376**	**0.61 (0.51–0.73)**
						
Ipilimumab + Chemotherapy	28	481	2.43 (1.63–3.6)	54	1084	1.77 (1.33–2.35)
Tremelimumab + Chemotherapy	8	147	0.62 (0.23–1.68)	13	319	1.14 (0.14–9.04)
**CTLA-4 + Chemotherapy**	**36**	**628**	**2.36 (1.67–3.35)**	**67**	**1403**	**1.70 (1.31–2.20)**
						
**ICI(s) + Chemotherapy**	**274**	**5292**	**1.35 (1.18–1.52)**	**537**	**13,733**	**1.39 (1.27–1.53)**

In the 3,450 ICI-related pneumonitis ICSRs from the VigiBase, a total of 274 reports where chemotherapy had been used were found. Among the 8 drug combinations investigated, ICSRs of 7 combinations (except cemiplimab plus chemotherapy combination due to the lack of data) were involved for pneumonitis. Overall, we identified that ICIs plus chemotherapy combination demonstrated a significant association with pneumonitis [ROR 1.35, 95% CI 1.18-1.52]. Among all the ICIs, anti-PD-1 antibodies combinations and anti-CTLA-4 antibodies combinations demonstrated an association with pneumonitis [PD-1+chemotherapy: 1.76, 95% CI 1.52-2.05; CTLA-4+chemotherapy: 2.36, 95% CI 1.67-3.35], while interestingly anti-PD-L1 antibodies plus chemotherapy combinations did not show the association. In sub-group analysis, we found a significant disproportionality signal for pneumonitis in three combinations [nivolumab + chemotherapy: 1.50, 95% CI 1.18-1.91, pembrolizumab + chemotherapy: 1.89, 95% CI 1.56-2.30; ipilimumab + chemotherapy: 2.43, 95% CI 1.63-3.60]. The combination with the strongest association is the ipilimumab plus chemotherapy combination.

In FAERS, similar trends were noted. In the 3,278 ICI-related pneumonitis ICSRs from the FAERS database, a total of 537 reports in which chemotherapy had been used were captured. All 8 drugs combination ICSRs reported pneumonitis. A significant disproportionality signal for pneumonitis was detected in ICIs plus chemotherapy combinations [ROR 1.39, 95% CI 1.27-1.53]. Similarly, the PD-1/CTLA-4 plus chemotherapy combination demonstrated an association with pneumonitis [PD-1+chemotherapy: 1.70, 95% CI 1.52-1.91; CTLA-4+chemotherapy: 1.70, 95% CI 1.31-2.20], while PD-L1 combination ICSRs did not show the association. Consistent with VigiBase data, nivolumab, pembrolizumab, and ipilimumab plus chemotherapy combinations demonstrated significant disproportionality signals [nivolumab + chemotherapy: 1.42, 95% CI 1.19-1.69, pembrolizumab + chemotherapy: 1.81, 95% CI 1.55-2.12; ipilimumab + chemotherapy: 1.77, 95% CI 1.33-2.35], and the pembrolizumab combination showed the strongest signals in FAERS database.

## 4 Discussion

A combination of ICI plus chemotherapy has now become the first-line therapy for multiple cancers. In various clinical trials (Chung et al., 2021; Emens et al., 2021; Janjigian et al., 2021), we have already known pneumonitis toxicities in the combination of ICI plus chemotherapy will be increased compared to chemotherapy alone. For example, the incidence of any-grade pneumonitis increased threefold in the pembrolizumab plus chemotherapy group compared with the chemotherapy group (Paz-Ares et al., 2018). However, whether the ICI treatment with antineoplastic drugs (before or concomitant with ICIs) increases the incidence of pneumonitis compared to other ICI-containing regimens in the real world remains debated. This is critically important in selecting a treatment regimen for patients with various clinical backgrounds, especially when determining the candidate patients for combination therapy. To the best of our knowledge, we report the first, largest, and most comprehensive analysis of the risk of pneumonitis in the combination of immune checkpoint inhibitors plus chemotherapy. In this study, we found a significant disproportionality signal in the combination of ICI plus chemotherapy compared to other ICI-containing regimens, including monotherapy. Different classes of ICI plus chemotherapy combinations showed distinct patterns.

We consistently found an increased reporting of pneumonitis toxicity for anti-PD-1/CTLA-4 plus chemotherapy compared with other anti-PD-1/CTLA-4 regimes in the two pharmacovigilance databases. This finding may support that there may be an increased risk of pneumonitis toxicity in an anti-PD-1/CTLA-4 plus chemotherapy combination. Consistent with our results, in a large network meta-analysis involving ICIs for multiple cancer types, Xu and colleagues (Xu et al., 2018) first identified that the risk of pneumonitis in ICIs with conventional therapies (chemotherapy, targeted therapy, and their combinations) is higher than the ICIs monotherapy alone. They also reported that anti-CTLA-4 drugs are associated with higher toxicities than anti-PD-L1 drugs. Contrary to the network meta-analysis from Xu and our findings, a network meta-analysis concerning lung cancer reported by Chen and colleagues found that the combination of ICI plus chemotherapy decreased the risk of grade 1–5 pneumonitis compared with ICI monotherapy and dual ICI combination (Chen et al., 2022). The reason for the contradiction might be explained by the types of cancers included in these studies, different data nature, and distinct control groups. The data from Chen’s study were only from lung cancer clinical trials and their conclusion were based on the comparison of ICI monotherapy and ICI combination, while we included data in various cancers and used all ICI-containing regimens (except for ICI plus chemotherapy) as the control group. A meta-analysis comparing the efficacy and safety of PD-L1+Chemo vs. PD-1+Chemo in extensive-stage small cell lung cancer revealed that PD-L1+Chemo tended to reduce the incidence of pneumonia of any grade, compared with PD-1+Chemotherapy (Yu et al., 2022). Similar to observations from previous studies, we found that the anti-PD-L1 plus chemotherapy combination was associated with less risk of pneumonitis, compared to other anti-PD-L1 containing regimens.

The age distribution of CIP patients exhibited a consistent pattern across both the VigiBase and FAERS databases, with the majority of cases concentrated within the 45-64 and 65-74 age brackets. In both databases, the 45-64 age group constituted the largest proportion of CIP patients, accounting for 25.04% and 28.68% in the VigiBase and FAERS databases, respectively. Among these patients, 24.37% and 27.00% were treated with ICI monotherapy, while 32.85% and 37.24% received a combination of ICI and chemotherapy in the VigiBase and FAERS databases, respectively. Subsequently, the 65-74 age group represented the second-largest proportion of CIP patients, with 23.97% and 26.94% in the VigiBase and FAERS databases, respectively. Within this demographic, 23.77% and 25.83% of CIP patients underwent ICI monotherapy, and 26.28% and 32.59% were treated with a combination of ICI and chemotherapy in the VigiBase and FAERS databases, respectively. These findings suggest a noteworthy consistency in age distribution and treatment modalities for CIP patients across both databases, emphasizing the importance of considering age-related factors in the management of CIP.

We also investigated the time to onset of CIP. We found the median time to CIP occurred early after therapy onset, either immunotherapy alone or in combination with chemotherapy (41 days vs. 37 days in FAERS, *p* < 0.05; 61.5 days vs. 74 days in VigiBase, *p* > 0.05). The median time of CIP onset in all ICI with or without chemotherapy is 62 days and 40 days from VigiBase and FAERS respectively. Similar to our findings, the time to onset of 315 lung cancer patients who predominantly received nivolumab or pembrolizumab at six centers in North Carolina is 52.5 days (Atchley et al., 2021), which is early occurred. A previous study reported the median time to the onset of CIP was typically approximately 2.8 months (the overall range spanned from 9 days to 19.2 months) from patients who received anti-PD-1/PD-L1 monotherapy or in combination with anti-CTLA4 therapy were identified at two institutions (Memorial Sloan Kettering Cancer Center: advanced solid cancers, 2009 to 2014, and Melanoma Institute of Australia: melanomas only, 2013–2015) (Naidoo et al., 2017). From a retrospective study of 205 patients with advanced NSCLC from Johns Hopkins Hospital who were treated with anti-PD-1/PD-L1 between 2007 and 2017, the median onset time of CIP was 82 days (Suresh et al., 2018). Another study retrospectively reviewed 160 consecutive patients who were diagnosed with NSCLC and treated with ICIs plus chemotherapy at Aichi Cancer Center Hospital between December 2018 and November 2020 indicated the median onset time of pneumonitis was 19.3 weeks (range: 1.6–108.9 weeks), whereas the median onset time of pneumonitis was 21.8 weeks (range, 1.6–108.9 weeks) in patients who received pembrolizumab and platinum plus pemetrexed (Yamaguchi et al., 2022). The difference in time on set may be due to the variety in the severity of patients. From the study of Zhang and colleagues, early-onset CIP caused death in patients, whereas no patients died of late-onset CIP (Moda et al., 2020). This may imply that early-onset CIP (within 3 months) may be more severe than late-onset CIP. Therefore, early-onset CIP has a greater impact on a patient’s clinical course and should be managed with greater care.

The precise potential mechanism driving ICI-related pneumonitis remains unclear. Postow et al. suggested that the occurrence of adverse events may be related to enhanced T cell activity against cross-antigens expressed in tumor and normal tissues (Postow et al., 2018). The pathogenesis of CIP may be caused by cytotoxic antigen-directed T-cell response (Zhai et al., 2020). Suresh et al. found that the bronchoalveolar lavage samples from CIP patients were mainly composed of CD4^+^ T cells (Tahir et al., 2019). Importantly, decreased CTLA-4 and PD-1 expression were observed in the regulatory T cell population, which suggested that increasing activated alveolar T cells and attenuating the anti-inflammatory Treg cells may lead to dysregulation of T cell activity (Suresh et al., 2019b). Besides, pre-existing autoantibodies may be potentially linked to the development of adverse events. Tahir et al. identified that the anti-CD74 level in the plasma of patients with CIP was elevated. Interestingly, overexpression of CD74 was also observed in samples of viral-mediated interstitial pneumonitis (de Souza Costa et al., 2014). Therefore, overexpression of CD74 may be a pathogenic nidus for CIP development. Additional exploration is required to deepen our understanding of CIP in cancer. In addition, an increase in the level of inflammatory cytokines is also related to the pathophysiology of immune-related adverse events. One study identified elevated levels of C-reactive protein and interleukin-6 in patients with atezolizumab-induced CIP when compared with baseline levels (Abdul Rafeh Naqash LVYSanderlin et al., 2018). Chemotherapy can alter the composition of the tumor immune microenvironment. Ende et al. reported that remodeling of TIM was observed in several tumor types after chemotherapy, including an increased level of CD3 or CD8 lymphocytes and decreased regulatory T cells (van den Ende et al., 2020). In addition, tumor cells killed by chemotherapy will release tumor antigens which may increase T cell activity and inflammatory cytokines levels (Heinhuis et al., 2019). The PD-L1 inhibitor could inhibit only the binding of PD-1 to PD-L1, while the PD-1 inhibitor could block the binding of PD-1 to both PD-L1 and PD-L2 (Chen and Han, 2015). Inhibition of PD-1 and PD-L2 interaction may also participate in the activation of T cells. This may explain why anti-PD-1 antibody combinations have a higher risk of CIP than anti-PD-L1 antibody combinations.

Although the reported incidence of CIP is rare (Zhu et al., 2020), restrictive enrollment criteria may underestimate the true incidence in clinical practice. Real-world experiences suggest the incidence of CIP is higher than clinical practice reported (Cho et al., 2018). CIP may result in wide-ranging respiratory symptoms with pulmonary parenchymal abnormalities and can progress to respiratory failure and death (Darnell et al., 2020). The diagnosis of CIP is typically after the exclusion of infection, tumor progression, and radiation-related pneumonitis (Martins et al., 2019). Presenting symptoms of CIP are variable and non-specific (Zhang et al., 2021), the most common concomitant toxicities are dyspnea, fatigue, pyrexia, and colitis in both VigiBase and FAERS databases. irAEs in other organs involve the skin, gastrointestinal tract, liver and endocrine organs (Salem et al., 2018; Bai et al., 2020; Zhou et al., 2022). Emerging researches suggest that the gut microbiota, a key factor in maintaining immune homeostasis, may affect the response and toxicity to checkpoint blockade therapy (Vetizou et al., 2015). Routy et al. showed that antibiotic consumption was associated with poor response to PD-1 blockade (Routy et al., 2018), However, the role of the microbiome in the development of ICI-driven irAEs remains unclear.

Our findings should be interpreted in the context of several limitations. First, pneumonitis may not have been uniformly defined over various geographic locations, but we carried out this analysis under the assumption that the potential differences are similar in each country. Second, pneumonitis ADRs in VigiBase and FAERS are mainly reported by health professionals and other non-health professionals spontaneously. Severe adverse events may be reported more frequently because people are more likely to volitionally report if an event is clinically significant. Third, although our study is the largest real-world analysis to date, it is retrospective in nature and therefore limited in its ability to establish causality. Our results should be considered hypothesis-generating, which provides useful insights into the potential associations. However, further research using real-world data is needed to validate our findings. Additionally, future studies should consider potential confounding factors, including disease severity, smoking status, and history of interstitial lung disease, to establish a more rigorous causal relationship. Last, reporters may only submit ICSR with complete concomitant medication information when they feel the information is relevant and not all ICSRs have complete concurrent medications data. The incomplete concomitant medication information may compromise the validity of our study results, but this influence was reduced by using the ROR method because we assume the incomplete concomitant medication information could happen in both cases and non-cases similarly. Despite these limitations, our study provides valuable insights into the potential risks associated with combining ICIs and chemotherapy and highlights the need for further research to fully understand the impact of this combination therapy on patients.

## 5 Conclusion

Based on the two worldwide largest adverse reaction databases, our worldwide pharmacovigilance analysis detected that the combination of ICI plus chemotherapy is significantly associated with pneumonitis toxicity compared to other ICI-containing regimens (including ICI monotherapy). Anti-PD-1 and anti-CTLA medications with additional chemotherapy could increase the risk of pneumonitis, whereas the risk was lower in the combination of anti-PD-L1 plus chemotherapy. Understanding the mechanisms underlying these interactions is critically needed. Additional studies to further confirm the detected association signal and to standardize guidelines for pneumonitis monitoring in ICI combination strategy should be conducted, particularly for anti-PD-1/CTLA-4 medications.

## Data Availability

The original contributions presented in the study are included in the article/Supplementary Material, further inquiries can be directed to the corresponding authors.
