# Supplementary material for: Comparison of pneumonitis risk between immunotherapy alone and in combination with chemotherapy: an observational, retrospective pharmacovigilance study
Source: Front Pharmacol. 2023 Apr 13;14:1142016. doi: 10.3389/fphar.2023.1142016 (PMC10133569; doi:10.3389/fphar.2023.1142016)
Supplement: Supplementary file 1 [file Table1.DOCX]

**Appendix**

Table S1. 76 cytotoxic chemotherapeutic drugs were selected as chemotherapy in this study.

|  | INN | Brand name |
| --- | --- | --- |
| 1 | Fluorouracil | 5-Fluorouracil |
| 2 | Capecitabine | Xeloda |
| 3 | Trifluridine | Lonsurf |
| 4 | Gemcitabine | Gemcitabine |
| 5 | Cytarabine | Cytarabine |
| 6 | Fludarabine | Fludara |
| 7 | Cladribine | Leustatin |
| 8 | Clofarabine | Clolar |
| 9 | Nelarabine | Arranon |
| 10 | Methotrexate | Methotrexate |
| 11 | Pralatrexate | Folotyn |
| 12 | Pemetrexed | Alimta |
| 13 | Raltitrexed | Tomudex |
| 14 | Azacitidine | Vidaza |
| 15 | Decitabine | Dacogen |
| 16 | Cisplatin | Cisplatin |
| 17 | Oxaliplatin | Eloxatin |
| 18 | Carboplatin | Paraplatin |
| 19 | Nedaplatin | Aqupla |
| 20 | Lobaplatin | Lobaplatin |
| 21 | Miriplatin | Miripla |
| 22 | Cyclophosphamide | Cytoxan |
| 23 | Ifosfamide | Ifex |
| 24 | Trofosfamide | Ixoten |
| 25 | Carmustine | Gliadel |
| 26 | Nimustine | Nidran |
| 27 | Fotemustine | Muphoran |
| 28 | Lomustine | CeeNU |
| 29 | Semustine | Me-CCNU |
| 30 | Ranimustine | Cymerine |
| 31 | Streptozocin | Zanosar |
| 32 | Chlormethine | Valchlor |
| 33 | Bendamustine | Treanda |
| 34 | Chlorambucil | Leukeran |
| 35 | Melphalan | Alkeran |
| 36 | Estramustine | Emcyt |
| 37 | Busulfan | Busilvex |
| 38 | Treosulfan | Trecondi |
| 39 | Thiotepa | Thioplex |
| 40 | Temozolomide | Temodal |
| 41 | Mitomycin | Mutamycin |
| 42 | Procarbazine | Procarbazine |
| 43 | Dacarbazine | Dacarbazine |
| 44 | Altretamine | Hexalen |
| 45 | Trabectedin | Yondelis |
| 46 | Bleomycin | Blenoxane |
| 47 | Peplomycin | Pepleo |
| 48 | Plicamycin | Plicamycin |
| 49 | Dactinomycin | Cosmegen |
| 50 | Amsacrine | Amsidine |
| 51 | Irinotecan | Campto |
| 52 | Topotecan | Hycamtin |
| 53 | Belotecan | Camtobell |
| 54 | Etoposide | Etoposide (Etopophos) |
| 55 | Teniposide | Vumon |
| 56 | Doxorubicin | Adriamycin |
| 57 | Epirubicin | Pharmorubicin |
| 58 | Idarubicin | Idamycin |
| 59 | Daunorubicin | Cerubidin |
| 60 | Pirarubicin | Pinorubicin |
| 61 | Amrubicin | Calsed |
| 62 | Valrubicin | Valstar |
| 63 | Aclarubicin | Aclacin |
| 64 | Mitoxantrone | Novantrone |
| 65 | Pixantrone | Pixuvri |
| 66 | Vinorelbine | Navelbine |
| 67 | Vindesine | Eldisine |
| 68 | Vinblastine | Velban |
| 69 | Vincristine | Oncovin |
| 70 | Vinflunine | Javlor |
| 71 | Paclitaxel | Taxol |
| 72 | Docetaxel | Taxotere |
| 73 | Cabazitaxel | Jevtana |
| 74 | Ixabepilone | Ixempra |
| 75 | Eribulin | Halaven |
| 76 | Ingenol | Picato |

INN: International nonproprietary name
